# A two-phase clustering procedure based on allele specific expression

**DOI:** 10.1186/s12859-026-06398-z

**Published:** 2026-04-01

**Authors:** Roberto Pagliarini, Francesco Nascimben, Alberto Policriti

**Affiliations:** Department of Mathematics, Computer Science, and Physics, University of Udine, Via delle Scienze 206, 33100 Udine, Italy

**Keywords:** Allele specific expression analysis, Spectral clustering, Unsupervised clustering, *Cis*-regulatory diversity

## Abstract

**Background:**

Allele Specific Expression analysis is an important tool for integrating genome and transcriptome data. It quantifies expression variation between the two haplotypes of a diploid individual distinguished by heterozygous sites, and is a powerful tool to estimate *cis-*regulatory diversity of alleles. Clustering algorithms can be used to identify patterns or groups of genes/samples based on their expression profiles. Depending on the structure of the data, different existing clustering algorithm can be adapted to allele specific expression data. However, no *ad-hoc* procedure has been developed.

**Results:**

In this work, we begin defining an expression matrix capturing allele expressions from an RNA-sequencing experiment. On this matrix, we develop a novel two-phase unsupervised clustering procedure, built on top of a spectral clustering algorithm, whose aim is to partition the population into groups of similar individuals, according to their allelic expression. As case-studies, the approach is used to cluster 98 cultivars representative of the variability observed in Vitis vinifera, starting from read counts of genes of chromosome 1 of leaves, and to analyze allele-specific count data from a CASTxMRL F1 hybrid mice dataset.

**Conclusion:**

Using the above mentioned real case-studies as well as generated synthetic data, we see that our algorithm shows significant robustness and outperforms other standard clustering techniques.

## Background

Clustering methods are unsupervised pattern classification algorithms that partition a data input space into groups. Their goal is to compute a *partition* (i.e. a subdivision in non-overlapping *classes* or *clusters*) where elements within the same cluster are similar, with respect to some collection of parameters, while elements in different clusters are dissimilar, with respect to the same collection of parameters.

Allele Specific Expression (ASE) analysis aims to integrate genome and transcriptome data, with the final goal of quantifying expression variation between the two haplotypes of a diploid individual distinguished by heterozygous sites [1]. Standard RNA-sequencing (RNA-seq) techniques capture differentially expressed alleles only when higher expression of one parental allele is shared between individual cells, as opposed to random mono-allelic expression of single cells that typically cancels out when a pool of poly-clonal cells is analyzed [2]. ASE analysis is often applied in studies of genetic regulation, where the expression levels of alleles inherited from each parent are measured independently. In ASE analysis clustering algorithms are not typically a core component of detecting ASE *per se*, but they are used to identify groups of genes or samples that share similar patterns of allele-specific expression. By clustering ASE data, it is possible to gain insights into regulatory mechanisms, identify gene networks, and detect potentially interesting genetic loci associated with specific phenotypes or diseases. Usually, standard clustering algorithms like hierarchical clustering, *k*-means, or more sophisticated methods like biclustering or consensus clustering are applied. Recently, *k*-means has been employed to study the ASE changes during T cell activation in autoimmune loci in [3], while hierarchical clustering analysis has been applied in [4] to analyze ASE of two maize hybrids, and in [5] to identify regulatory variants underlying complex traits in cattle. In recent ASE analysis, hierarchical clustering has also been extended by principal component analysis [6, 7]. Clustering based on Gaussian mixture and *k*-means models has also become an integral part of bioinformatic pipelines for ASE analysis in single cells [8–10]. Unfortunately, traditional clustering algorithms often struggle to handle the complex, high-dimensional data and diverse distributions present in biological data. Due to this heterogeneity, they may fail to identify meaningful clusters [11].

We have recently developed a new computational approach in order to quantify allele specific expression at population level [12]. ASE clustering across different populations can reveal population-specific regulatory variants, providing insights into how genetic diversity contributes to phenotypic variation. The main challenge is the biological heterogeneity across individuals that can reduce the performances of the classical clustering algorithms, particularly in complex tissues.

In this work, we introduce an expression matrix capturing allele expression provided by an RNA-seq experiment. On this matrix, we develop a novel two-phase unsupervised clustering procedure based on the *spectral clustering* algorithm [13], with the goal of partitioning the population into groups of similar individuals, according to their allelic expression. Spectral clustering is a graph-based algorithm for finding *k* arbitrarily shaped clusters in data. The technique involves representing the data in a lower-dimensional space, so that data clusters are more widely separated, allowing us to use algorithms such as *k*-means or *k*-medoids clustering. This lower dimension is based on the eigenvectors of a Laplacian matrix, derived by modeling the local neighborhood relationships between data points as an undirected similarity graph. In order to apply spectral clustering, the number *k* of clusters must be specified beforehand: in this paper, we will also analyze several ways to estimate the optimal number of clusters for our ASE dataset. Particularly, at both phases the proposed algorithm computes a new representation of the individuals, starting from a suitably-defined similarity matrix and through a *spectral decomposition* process, which can be used to identify meaningful clusters. While our approach builds upon well-established methodologies, its novelty lies in the integrated analysis of the amount of shared alleles across individuals within a population, followed by the examination of their allele-specific expression profiles.

As a first case study, we will show the application of our method to a population of 98 cultivars representative of the variability present in Vitis vinifera, starting from ASE of genes belonging to chromosome 1 of leaves obtained from [14]. It is important to point out that a set of 1/19 of genes represents a random sample even if it corresponds to a single chromosome. This is due to the fact that there are many more differences within a chromosome then between different chromosomes that can impact on allele imbalance, such as, for instance, chromatin state or gene expression levels. We will then evaluate the robustness of the proposed algorithm by employing synthetic data. Then, as a second case study, we will consider the ASE dataset generated in [6] to study tissue regeneration in mice.

Everything we will discuss in the paper has been implemented in MATLAB®[15].

The paper follows this structure: in Sect. 2, we describe our input data and the expression matrix capturing it; we then provide a short excursus on the spectral clustering algorithm, before going into the details of our novel procedure. In Sect.3, we describe our dataset and we analyze the experimental results. In  Sect. 4 an analysis of the time complexity of our algorithm is reported. Finally, in  Sect. 6 we draw conclusions and discuss possible improvements of the method, which we plan to further investigate in the future.

## Methods

### Allelic expression input data

Let us consider a set of *n* genes $G= \{g_1, \dots , g_n \}$ and a population of *m* heterozygous individuals $P= \{p_1, \dots , p_m \}$, such that each gene is expressed in at least one individual. We assume a gene $g_i \in G$ to be expressed, among the whole population, by a set of *a*(*i*) distinct alleles $\mathcal {A}^{g_i}= \{A^{g_i}_{1},A^{g_i}_{2}, \dots , A^{g_i}_{a(i)} \}$. Then, given any $g \in G$, the RNA-seq experiment provides, for each individual $p \in P$, the alleles representing the genotype of *g* in *p* (if any), along with their corresponding read counts. In the following, we will use “read counts” and “expression level” synonymously, although the latter may sometimes have a different meaning in the literature.

Let $a = \sum _{i=1}^{n}a(i)$ be the total number of alleles observed in the RNA-seq experiment. We define *expression matrix* $\textbf{E} \in \mathbb {N}^{a \times m}$, containing read counts for each individual: more precisely, for a given column associated to an individual $p_i$, the first *a*(1) rows represent $g_1$-alleles expression levels in $p_i$, the following *a*(2) rows represent $g_2$-alleles expression levels in $p_i$ and so on. It holds that, for any given individual $p_i$ and any given gene *g*, the number of non-null elements in the subsection of the *i*-th column associated to *g* can be either 0 (if $p_i$ does not express *g*), 1 (homozygous individual) or 2 (heterozygous individual): this yields a very sparse expression matrix, with at most 2*mn* out of the total *am* elements of $\textbf{E}$ possibly being greater than 0.

What we have described so far refers to data obtained from a single chromosome on a single tissue (e.g. leaves) of the population. Since RNA-seq experiments typically involve a number of chromosomes and tissues, a distinct expression matrix could be generated and processed for each chromosome-tissue combination. Depending on the application scenario, a matrix featuring read counts from more than one such combination could also be easily built, by vertical concatenation of distinct expression matrices.

### The spectral clustering algorithm

The *spectral clustering* algorithm [13] represents the building block of our two-phase clustering procedure. Given a collection of *m* data points $P= \{p_1, p_2, \dots , p_m \}$ and an *m*-by-*m* matrix $\textbf{S}$ such that $s_{ij}$ is a *similarity measure* between two points $p_i$ and $p_j$, spectral clustering aims to partition *P* into a given number *k* of clusters, in order to maximise and minimise intra- and inter-cluster similarities, respectively.

More specifically, spectral clustering partitions into *k* classes the *similarity graph*: a weighted graph $G = (P,E,\textbf{W})$, with $P$ as nodes, $E$ edges, and $\textbf{W}$ representing weights. Several constructions can be used to define how nodes in *G* should be connected through $E$: the simplest one is the fully connected construction, where $\{p_i, p_j\} \in E$ if and only if $s_{ij} > 0$.

Whatever the construction of choice, the weight matrix $\textbf{W}$ is defined so that $w_{ij}= s_{ij}$ if $\{p_i, p_j\} \in E$ and $w_{ij}= 0$ otherwise. Let $\textbf{D}$ be the *degree* matrix of *G*, i.e. the diagonal matrix where:

1 $$\begin{aligned} d_{ii}= \sum _{j=1}^{m} w_{ij}. \end{aligned}$$

The *unnormalised graph Laplacian* of *G* is thus defined as $\textbf{L}= \textbf{D} - \textbf{W}$. An overview over many of the features of the unnormalised Laplacian matrix can be found in [16, 17]. In our approach, we employed the *normalized random-walk Laplacian matrix* [18] defined as:

2 $$\begin{aligned} \mathbf {L_{rw} = \textbf{D}^{-1}\textbf{L}} \end{aligned}$$

and then the following generalized eigenvalue problem

3 $$\begin{aligned} L v = \lambda D v \end{aligned}$$

is solved to derive $\mathbf {L_{rw}}$, where *v* is a column vector of length *m* and $\lambda$ is a scalar. The values of $\lambda$ that satisfy the Eq. (3) are the generalized eigenvalues of matrix (2).

We solved the generalized eigenvalue problem (3) by applying the algorithm developed in [19]. Let $\{u_1, u_2, \dots , u_k\}$ be the first *k* eigenvectors of $\mathbf {L_{rw}}$, i.e. the eigenvectors associated to its *k* smallest eigenvalues, and let $\textbf{U} \in \mathbb {R}^{m \times k}$ the matrix having said eigenvectors as columns. Furthermore, let $Y= \{y_1, y_2, \dots , y_m \}$ the set of $\mathbb {R}^k$ vectors corresponding to the rows of $\textbf{U}$: $y_i \in Y$ defines a new representation of the corresponding data point $p_i \in P$. Finally, by applying *k*-means on *Y*, the procedure obtains a partitioning of the original dataset *P* into *k* classes, such that intra-cluster distance, i.e. the sum of all squared euclidean distances between a data point and the centroid (*mean*) of the cluster it belongs to, is minimised. After randomly selecting *k* initial centroids, the procedure iteratively assigns all data points to the cluster associated to their closest centroid and recomputes all means, stopping as soon as the cluster assignments no longer change. Since the choice of the initial centroids may influence the end result, the algorithm is usually run a number of times, keeping only the best partition.

The meaningfulness of the method lies in the fact that weights in the similarity matrix can be seen as likelihoods of moving from one node in *G* to another in a single step: if clusters of highly similar nodes exist, the probability of starting from a node in such a cluster and ending up outside of it after *t* steps, with *t* a sufficiently high number, will be extremely low. *Y*, the eigenvector-based representation of *P*, captures this phenomenon.

### Recovering the optimal number of clusters

Spectral clustering requires the number *k* of clusters to be specified beforehand. In scenarios in which this number is not known, a solution is to run a given clustering algorithm using different values for *k* and then compare the obtained results afterwards. This process is called *cluster validation* and its aim is the analysis of how well a partition fits the structure underlying the data [20]. A number of cluster validity indices could be used to estimate *k*: we have implemented in our procedure a few indices, among the most popular, that work with general distance measures. They are the *Calinski–Harabasz index (CHI)* [21], the *Davies-Bouldin index (DBI)* [22], the *Silhouette criterion (SHI)* [23], and the *Dunn index (DUI)* [24], which we introduce below.

Let us consider our set *P* of *m* individuals. Let us define: *c* as the center of *P* (i.e. its average point in vector space $\mathbb {R}^{a}$), $N_{\mathcal {C}}$ as the number of clusters, $\mathcal {C}_{i}$ as the i-th cluster, $n_{i}$ as the cardinality of $\mathcal {C}_{i}$, $c_{i}$ as the center of $\mathcal {C}_{i}$, and *d*(*x*, *y*) as the Euclidean distance between *x* and *y*. To simplify notation, in the following we assume indices *i* and *j* to always vary within integer range $[1..N_{\mathcal {C}}]$. The Silhoutte index validates clustering performance considering the pairwise difference of between- and within-clusters distances, that is:

4 $$\begin{aligned} SHI = \frac{1}{N_{\mathcal {C}}}\sum _{i}\left\{ \frac{1}{n_{i}}\sum _{x \in \mathcal {C}_{i}}\left\{ \frac{b(x)-a(x)}{max\{a(x),b(x) \}}\right\} \right\} \end{aligned}$$

where *a*(*x*) is the average dissimilarity of *x* to all elements of $P \setminus \{ x \}$, and *b*(*x*) the minimum average dissimilarity between *x* and all clusters $\mathcal {C}_{i}$ such that $x \notin \mathcal {C}_{i}$. $SHI \in [-1,1]$, and the optimal cluster number is determined by maximizing this index.

The Dunn index is defined as the ratio of the smallest distance between clusters, which estimates the separation of groups, and the maximum cluster diameter, that estimates its cohesion, namely:

5 $$\begin{aligned} DUI = \min _{i}\left\{ \min _{j, j \ne i} \left\{ \frac{\min _{x \in \mathcal {C}_{i},y \in \mathcal {C}_{j}}d(x,y)}{\max _{k}\left\{ \max _{x,y \in \mathcal {C}_{k}} d(x,y)\right\} }\right\} \right\} . \end{aligned}$$

The Calinski-Harabasz index evaluates cluster validity based on the average between- and within-cluster square distances, i. e.:

6 $$\begin{aligned} CHI = \frac{\sum _{i}\left\{ n_{i}d^{2}(c,c_{i})/ (N_{\mathcal {C}} - 1)\right\} }{\sum _{i}\sum _{x \in \mathcal {C}_{i}}\left\{ d^{2}(x,c_{i})/(m - N_{\mathcal {C}})\right\} }. \end{aligned}$$

For both indices (5) and (6) the optimal cluster number is determined by maximizing their values.

The Davis-Bouldin index is calculated in this way. For each cluster $\mathcal {C}$, the similarities between $\mathcal {C}$ and all the other clusters are computed, and the highest value is assigned to $\mathcal {C}$ as its cluster similarity. Then the DBI index is obtained by averaging all the cluster similarities.

7 $$\begin{aligned} DBI = \frac{1}{N_{\mathcal {C}}} \sum _{i} \max _{j, j \ne i} \left\{ \left[ \frac{1}{n_{i}} \sum _{x \in \mathcal {C}_{i}} d(x,c_{i}) + \frac{1}{n_{j}} \sum _{x \in \mathcal {C}_{j}} d(x,c_{j})\right] /d(c_{i},c_{j})\right\} . \end{aligned}$$

The smaller $DBI$ is, the better the clustering result. By minimizing this index, clusters are the most distinct from each other, therefore achieving the best partition.

### Two-phase spectral clustering

The proposed two-phase procedure is based on the formulation of spectral clustering described in 2.2, and its aim is to partition the population *P* of *m* individuals starting from ASE analysis. In the first phase, *P* is clustered according to the fraction of shared alleles between individuals; in the second one, clustering is further performed within each class resulting from the first phase, based on correlation of expression levels between individuals.

The rationale for this procedure is the following: by first partitioning the population according to the amount of allelic overlap, the resulting classes should contain individuals which, pairwise, share a non-negligible amount of alleles. Thus, further refinement of these classes based on expression levels should be more meaningful than simply clustering the whole population according to the same criterion since, in general, two randomly selected individuals only share a few alleles at most. To see why this is the case, let us focus on a specific allele in the following scenarios: in the first one, the allele is expressed in both $p_i$ and $p_j$, with approximately equal read counts; in the second one, the allele is not expressed in $p_i$ at all, meaning that the corresponding element of expression matrix $\textbf{E}$ is 0, while it is expressed in $p_j$, with a very low number of read counts. Although the numerical difference between $p_i$ and $p_j$ will be very limited in both cases, it seems quite reasonable that $p_i$ and $p_j$ should be considered more similar in the former scenario with respect to the latter. This example shows how mere comparison of read counts between $p_i$ and $p_j$ does not capture the genetic importance of an allele being expressed in one individual and not in the other: the preliminary clustering, based on the number of shared alleles, should compensate for (or, at least, mitigate) this lack of finesse.

During each phase, spectral clustering is performed starting from a similarity matrix. We use a modified version of the *k*-means algorithm, named *k*-means$++$, which has been shown to improve both the speed and the accuracy of the original *k*-means [25]. Although finding an exact solution to the *k*-means problem for arbitrary input is NP-hard [26], the standard approach to finding an approximate solution, known as Lloyd’s algorithm [27], is widely used and frequently finds reasonable solutions quickly. However, this approximation can be unboundedly worse than the optimal clustering with respect to the objective function. The *k*-means$++$ algorithm addresses this point by specifying a procedure to initialize the cluster centers before proceeding with the standard *k*-means optimization iterations.

In order to determine the optimal number of clusters at each step, one of the indices described in subsection 2.3 is employed. Aiming to achieve both a feasible time consumption and a realistic simulation, we choose the value *k* optimizing the selected index within interval [2, *m*/4], where *m* is the cardinality of the population. The upper bound of the interval has also been set in order to prevent the occurrence of too many minuscule clusters: besides, on our dataset, preliminary testing showed $\hat{k}$ to be between 2 and 10 in a vast majority of cases.

We now detail the specifics for each phase of the procedure. Let $A(p) \subseteq \bigcup _{i=1}^n \mathcal {A}^{g_i}$ be the set of alleles, among all considered genes, which are expressed in individual *p*: it holds that, for all individuals, $\vert A(p) \vert$ ranges between 0 (no gene is expressed in *p*) and 2*n* (all genes are heterozygously expressed). We recall that $\mathcal {A}^g$ is the set of alleles of gene *g* which are expressed at least once in the population.

#### Phase one

Initially, a cluster of all individuals for which no gene is expressed is created manually: we apply this filtering because one or more errors could occur during an RNA-seq experiment, leading to corrupted genotypes.

Then, we define *allelic-similarity* matrix $\textbf{S}^A \in \mathbb {N}^{m \times m}$, such that element $s^A_{ij}$ is the Jaccard index of sets $A(p_i)$ and $A(p_j)$:

8 $$\begin{aligned} s^A_{ij}= \frac{\vert A(p_i) \cap A(p_j) \vert }{\vert A(p_i) \cup A(p_j) \vert }. \end{aligned}$$

$s^A_{ij}$ is a similarity measure between $A(p_i)$ and $A(p_j)$: it ranges from 0, when $p_i$ and $p_j$ share no common allele, to 1, when $p_i$ and $p_j$ express exactly the same alleles.

The similarity graph $G=(P,E,\textbf{W})$ is built using the fully connected construction, i.e. having $\{p_i,p_j \} \in E$ if and only if $s^A_{ij} > 0$. Spectral clustering is then performed and the whole population *P* is partitioned into the optimal number of classes determined by the CHI index, as previously described.

#### Phase two

The following procedure is applied to each cluster obtained after the first phase. For the sake of explanation, let us now focus on one such class $\mathcal {P}$ and denote by $m'= \vert \mathcal {P} \vert$ its cardinality. We define *expression-similarity* matrix $\textbf{S}^E \in \mathbb {R}^{m' \times m'}$, whose element $s^E_{ij}$ is computed as the absolute value of the Pearson correlation [28] between columns *i* and *j* of expression matrix $\textbf{E}$. To build the similarity graph, we employ a slightly more restricted variant of the fully connected method, such that edge $\{p_i,p_j \} \in E$ if and only if $s^E_{ij} > 0$ *and* the correlation between $p_i$ and $p_j$ is statistically significant, i.e. if its *p*-value is smaller than 0.05, where *p*-values are computed by using the Student’s t distribution related to the correlation values. This correlation graph, on which we again apply spectral clustering, allows the modelling of individuals in modules that are the results of allele specific expression.

### Selecting the optimal solution

In the spectral clustering, the *k*-means algorithm is employed: it uses an iterative procedure to minimize the sum of point-to-centroid distances, over all *k* clusters. Firstly, *k*-means employs *batch updates*, where each iteration consists of reassigning points to their nearest cluster centroid, all at once, followed by recalculation of cluster centroids: this may occasionally not converge to a local minimum solution, i.e. a partition of the data where moving any single point to a different cluster increases the total sum of distances. This batch phase is fast, but potentially only approximates a solution. Therefore, in a second step, the algorithm uses *online updates*, where points are individually reassigned if doing so reduces the sum of distances, and cluster centroids are recomputed after each reassignment. Each iteration during this step consists of one pass though all the points. Operating in this way the algorithm converges to a *local* minimum.

We tackle this problem by running our two-phase clustering procedure several times and selecting the result that minimizes the *S-Dbw* index [29], an *internal validation* measure. Internal validation indices, based on intrinsic data information, evaluate a clustering result without relying on external information [30] and are thus useful to assess the quality of the generated partitions in scenarios in which class labels are not available, such as our case study. In [31] the authors investigated the validation properties of eleven internal clustering measures in five different aspects, finding that the *S-Dbw* index is the only one performing well in all the considered aspects, while others display limitations in several application scenarios. *S-Dbw* is the summation of data set compactness, measured by cluster variance, and of separation, obtained by the density between clusters: the minimum value indicates the best result. Formally, the formulation of *S-Dbw* is based on the Euclidean norm $\Vert x \Vert = \sqrt{(x^{T}x)}$, the standard deviation of a set *X* of object $\sigma (X) = \frac{1}{|X|}\sum _{i = 1}^{|X|}(x_{i} - \bar{x})^{2}$, and the standard deviation of a partition $\mathcal {C} = \{\mathcal {C}_{1},\mathcal {C}_{2},\ldots ,\mathcal {C}_{k}\}$, $stdev(\mathcal {C}) = \frac{1}{k}\sqrt{\sum _{i = 1}^{k}\Vert \sigma (\mathcal {C}_{i}) \Vert }$. Then,

9 $$\begin{aligned} S-Dbw(\mathcal {C}) = \frac{1}{k} \sum _{i = 1}^{k}\frac{\Vert \sigma (\mathcal {C}_{i})\Vert }{\Vert \sigma (X)\Vert } + \frac{1}{k(k-1)}\sum _{i = 1}^{k}\sum _{j = 1}^{k}\frac{den(\mathcal {C}_{i},\mathcal {C}_{j})}{\max \{den(\mathcal {C}_{i}),den(\mathcal {C}_{j})\}} \end{aligned}$$

where

10 $$\begin{aligned} den({\mathcal {C}_{i}}) = \sum _{x_{l} \in \mathcal {C}_{i}}f(x_{l},\bar{\mathcal {C}_{i}}) \end{aligned}$$

11 $$\begin{aligned} den({\mathcal {C}_{i}},{\mathcal {C}_{j}}) = \sum _{x_{l} \in \mathcal {C}_{i} \cup \mathcal {C}_{j}}f\left( x_{l},\frac{\bar{\mathcal {C}_{i}}+\bar{\mathcal {C}_{j}}}{2}\right) \end{aligned}$$

and

12 $$\begin{aligned} f(x_{l},\mathcal {C}_{i}) = \left\{ \begin{array}{rl} 0 & \text{ if } d(x_{l},\bar{\mathcal {C}_{i}})> stdev(\mathcal {C}) \\ 1 & \text{ otherwise. } \end{array} \right. \end{aligned}$$

## Results

### Grapevine variants dataset

A grapevine variants database previously developed has been used. Berries and leaves samples have been collected, used for library preparation, and sequenced [14]. RNA was extracted using the Spectrum Plant Total RNA Kit of Sigma-Aldrich. Approximately 500 ng of RNA was used for library construction with the TruSeq Stranded mRNA Kit of Illumina for leaf RNA and with the Universal Plus mRNA−Seq Library Preparation Kit (Tecan Genomics) for berry RNA. Paired-end reads were obtained from Illumina HiSeq2000 and HiSeq2500 sequencers. The raw reads have been filtered with ERNE−filter v.1.4.6 [32], to extract chloroplast reads and remove reads of low quality and shorter than 50 pb. The alignment of the reads to the reference genome of *Vitis vinifera* was performed with the software STAR v2.5 [33]. A quality assessment of the samples was performed, considering the percentage of read duplicates in the alignment and the outliers obtained with a principal component analysis of the aligned reads. SHAPEIT2 [34] has been used to infer the haplotypes of the samples by using a previously obtained set of SNPs. Then, the information of the phased SNPs have been integrated with the reference genome in FASTA format obtaining the sequences of the two haplotypes in all the cultivars. After that, the software ALLIM v1.1 [35] has been applied to estimate the allele-specific expression of the genes in all the tissues and cultivars.

As case-study, we conducted experiments on our dataset to cluster 98 cultivars representative of the variability present in *Vitis vinifera*, by employing read counts of genes in chromosome 1 of leaves.

### Cluster validation of the optimal partition indexes

In order to test our algorithm with different validation indexes, and their performances on allele specific expression data, we applied it to partition the ASE data of chromosome 1 of leaves by employing the criteria described in subsection 2.3.

To assess similarity among clustering results, we employed Adjusted Rand Index (ARI) [36]. Given clustering $\mathcal {C} = \{\mathcal {C}_{1},\mathcal {C}_{2},\ldots ,\mathcal {C}_{k}\}$, it is defined as:

13 $$\begin{aligned} ARI = \frac{\sum _{ij} \left( {\begin{array}{c}n_{ij}\\ 2\end{array}}\right) - \left[ \sum _{i} \left( {\begin{array}{c}a_{i}\\ 2\end{array}}\right) \sum _{j} \left( {\begin{array}{c}b_{j}\\ 2\end{array}}\right) \right] /\left( {\begin{array}{c}n\\ 2\end{array}}\right) }{\frac{1}{2}\left[ \sum _{i} \left( {\begin{array}{c}a_{i}\\ 2\end{array}}\right) + \sum _{j} \left( {\begin{array}{c}b_{j}\\ 2\end{array}}\right) \right] - \left[ \sum _{i} \left( {\begin{array}{c}a_{i}\\ 2\end{array}}\right) \sum _{j} \left( {\begin{array}{c}b_{j}\\ 2\end{array}}\right) \right] } \end{aligned}$$

where $n_{ij} = \vert \mathcal {C}_{i} \cap \mathcal {C}_{j} \vert$, $a_{i} = \sum _{j=1}^{k}\vert \mathcal {C}_{i} \cap \mathcal {C}_{j} \vert$, and $b_{i} = \sum _{i=1}^{k}\vert \mathcal {C}_{i} \cap \mathcal {C}_{j} \vert$. ARI, which ranges from $- 1$ to 1, is a measure, adjusted for the chance grouping of elements, of the similarity between two partitions of the same data, obtained by considering all pairs of samples and counting couples that are assigned to the same or different clusters. ARI thus tends to 0 for random labelings, independently of the number of clusters and samples, is exactly 1 when the partitions are identical, up to a permutation, while is $- 1$ for poor matches. Table 1 shows that the results obtained by applying SHI and DUI are comparable, while those obtained by the other validation indexes are very different.

**Table 1:** Experimental results. Similarity among the results of the three clustering algorithms, computed by adjusted rand index

	SHI	DUI	CHI	DBI
SHI	1.000	0.972	0.222	0.060
DUI	0.972	1.000	0.211	0.056
CHI	0.222	0.211	1.000	0.150
DBI	0.060	0.056	0.150	1.000

**Fig. 1:**
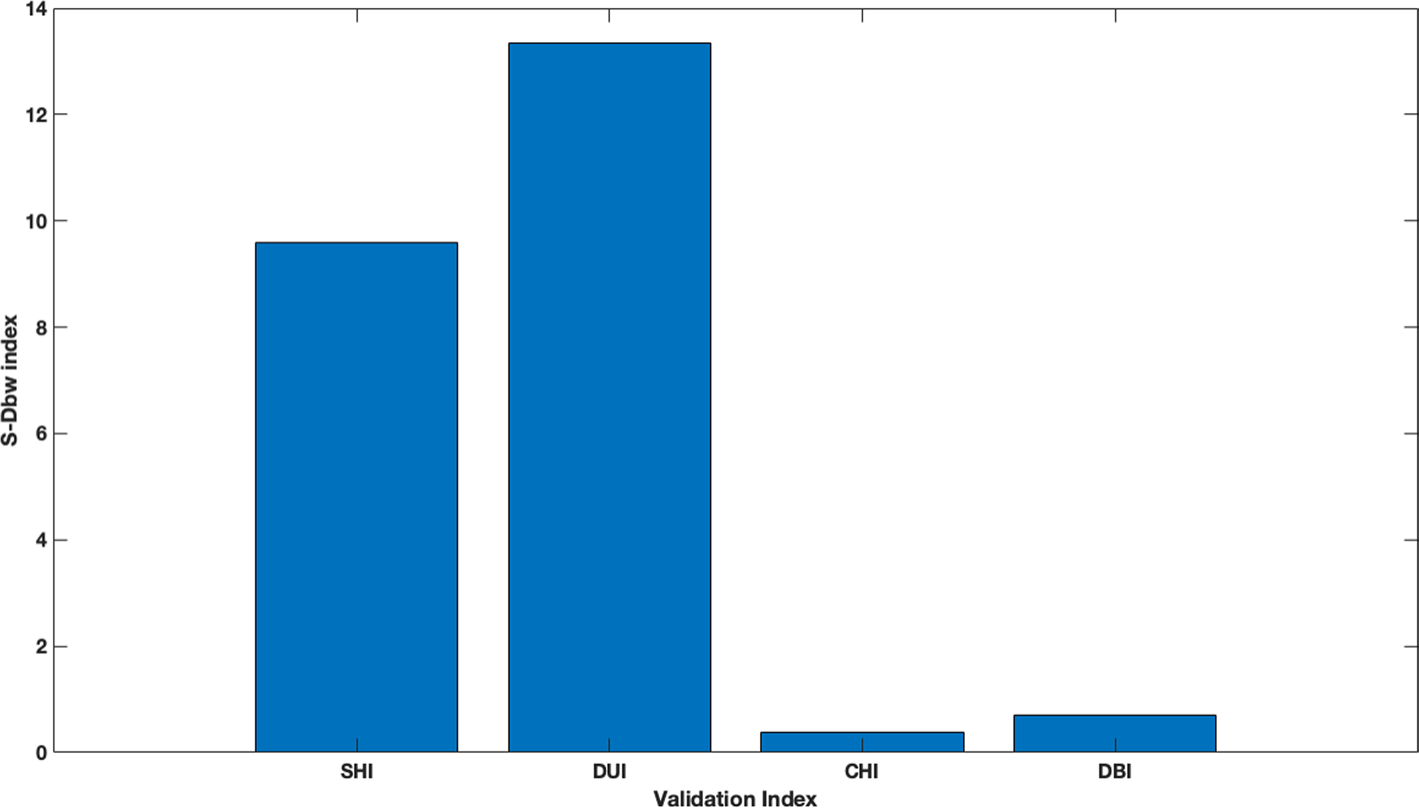
Validation of the combination of the proposed two-phase clustering algorithm with several validation indexes. *S-Dbw* index values computed for the clustering results obtained by applying our two-phase algorithm with the indexes described in subsection 2.3. We can observe that the minimum (best) value for *S-Dbw* index is obtained with CHI

Specifically, after applying the initial filter, our algorithm partitioned the population into 7 clusters when combined with SHI, into 5 groups with DUI, into 15 partitions with CHI, and into 23 clusters with DBI. Since class labels are not available in our application scenario, we resorted to *S-Dbw* index, described in subsection 2.5, to assess the quality of the generated partitions. Figure 1 shows that the best value was obtained by combining the proposed two-phase procedure with CHI, but application of DBI also leads to a comparable outcome. On the other hand, DUI and SHI gave us very poor outputs. Thus, we can observe that the validation indices linked to poor results are those that cluster individuals into a small number of groups.

We therefore analyzed the possible causes of these large differences in performances among the considered indexes. Each of them estimates cluster cohesion and cluster separation and combines them, in order to compute a quality measure. The combination is performed by a division or a sum [37]. Particularly, we can say that, for both CHI and DBI, intra-variance and inter-variance are based on *global computation*. Namely, CHI (6) is a ratio-type index where cohesion is estimated based on the distances between points of a cluster and their centroid, while separation is based on the distances between the centroids of each cluster and the centroid of the entire dataset. DBI (7) estimates intra-variance based on the distance from the points in a cluster to its centroid, while the inter-variance is computed by considering the distance between centroids. Differently, DUI (5) estimates cohesion by the nearest neighbour distance and separation by the maximum cluster diameter, while SHI (4) measures intra-variance by taking into account the distance between all the point in the same cluster and inter-variance by considering the neighbour distance. There are other SHI limitations to keep in mind. It can only be applied when the clusters have a spherical shape: if this is not the case, clustering quality cannot be realistically measured. For the same reason, SHI is sensitive to noise.

Due to the assumptions behind our algorithm, the impact of subclusters also has to be carefully analyzed. Internal cluster separation is supposed to have a sharp decline when partition number increases from the optimal *k* to $k+1$ [37]. SHI employs the average minimum distance between clusters as the inter-cluster separation. Thus, in the case of a dataset with subclusters, separation peaks when subclusters close to each other are considered as a unique partition: therefore, the wrong value of *k* (smaller than the optimal one) will be chosen. DUI uses the minimum pairwise distance between cluster centers to evaluate separation: in the case of a dataset with close subclusters, the same considerations discussed for SHI apply to DUI, which thus fails to compute the optimal value of *k* [31].

### Interpretation of the clustering result

Initially, a cluster has been identified by filtering three individuals for which no gene is expressed. Therefore, our two-phase clustering algorithm, considering CHI, partitioned the population into 15 clusters. In particular, two clusters, $\mathcal {C}_{1}$ and $\mathcal {C}_{2}$, were obtained after the first phase: in the second phase, members of $\mathcal {C}_{1}$ and $\mathcal {C}_{2}$ were further grouped into 7 and 8 sub-clusters, respectively. These results are available at the following URL: https://github.com/RobertoPagliarini/Two-phase-clustering-procedure/tree/main/ResultsCIBB2024. We found that the inferred groups preserve history, population structure, and geographic differentiation in cultivated grapes (Details of the grapes names can be found in the file VitisviniferaClusteringResuls.xlsx).

Cluster 1 includes varieties sharing conserved regulation of secondary metabolites and phenolic biosynthesis pathways. These influence berry skin color, tannin content, and mouthfeel–key wine quality traits [38, 39]. Cluster 2 contains a single cultivar, *Sciavtsitska*, a rare white grape variety indigenous to Georgia. Its isolation in a distinct group likely reflects a unique pattern of ASE compared to other cultivars in the dataset. This uniqueness may be attributed to: (*i*) its geographic origin, as Georgia is one of the primary centers of grapevine domestication and harbors a high degree of genetic and transcriptomic diversity; (*ii*) expression of distinct allelic variants in genes related to secondary metabolism, which are often highly variable across landraces and traditional cultivars [40]. Cluster 3 includes varieties that share several key biological and genetic traits that likely explain their grouping based on ASE. They exhibit strong expression of alleles involved in the flavonoid and anthocyanin pathways, which influence pigmentation, a trait often under cis-regulatory control. Moreover, some studies have shown that *Pinot Noir* and *Traminer* share haplotypes in genomic regions controlling aroma precursors, possibly maintained through human selection for similar wine profiles [41, 42]. Cluster 4 contains cultivars which share transcriptional and metabolic features. Both varieties in this group are late-ripening varieties known to develop their full aromatic potential under extended ripening periods, often in high-stress environments. This suggests similar ASE patterns in stress-responsive genes, especially those under cis-regulatory control [43, 44]. Cluster 5 comprises three cultivars that share key regulatory traits. They are associated with aromatic profiles rich in terpenes, thiols, and esters. These aromas are controlled by genes involved in monoterpene and thiol biosynthesis, where cis-regulatory variants may drive differential ASE patterns. Moreover, these varieties are well-suited to humid or maritime climates, often showing ASE in genes involved in abiotic stress response, that help maintain grape health and aroma precursor integrity during ripening. Another important observation is that the grapes in this cluster are often used in wines requiring aromatic freshness and structure, suggesting similar post-veraison gene regulation consistent with common ASE patterns [45, 46]. Cluster 6 consists solely of *Pecorino*, a white grape variety native to the Marche and Abruzzo regions of Italy, and well-known for its adaptation to warm, drought-prone environments, showing resilience through upregulation of stress-response genes such as dehydrins, heat shock proteins, and antioxidant enzymes. ASE signals in these gene sets highlight strong cis-regulatory variation influencing environmental adaptation. *Pecorino* exhibits a rich phenolic profile with considerable flavonoid and hydroxycinnamate accumulation. Moreover, genomic studies reveal that this cultivar carries alleles and regulatory variants uncommon among other wines, consistent with its solitary clustering [47]. Cluster 7 groups together *Cabernet Franc*, *Cabernet Sauvignon*, *Cesanese Daffile*, *Ogialesci*, *Rhein Riesling*, *Tschvediansis Tetra*, and two unidentified accessions labeled *V278* and *V411*. *Cabernet Franc* and *Cabernet Sauvignon* are genetically related cultivars. Their ASE similarity is expected due to shared haplotypes and regulatory variants. *Cesanese Daffile* and *Ogialesci* may share metabolic pathways linked to polyphenol biosynthesis and stress responses common in red grape varieties. Varieties in this group are often cultivated in temperate regions and show coordinated expression changes in abiotic and biotic stress-related genes, which influence berry development and resilience, reinforcing their shared ASE signatures [48]. Finally, the presence of two unidentified accessions clustered with known varieties suggests they may belong to genetically related or hybrid backgrounds exhibiting similar regulatory mechanisms. Varieties in cluster 8 exhibit distinctive aromatic and phenolic characteristics regulated by the biosynthesis of flavonoids pathways [49]. Morevoer, these varieties are traditionally grown in geographically proximate and climatically similar regions in Northern Italy. Therefore, ASE patterns may reflect adaptation-driven gene expression differences related to stress tolerance, such as responses to temperature fluctuations and pathogen resistance. Cluster 9 groups a diverse set of varieties sharing similar expression patterns in genes involved in fruit maturation, sugar metabolism, and acid degradation, which are key processes influencing berry quality traits such as sweetness, acidity, and texture. Many of these grapes originate from regions with challenging climates or environmental stressors. This cluster likely retains common expression regulation in secondary metabolism genes, particularly those involved in volatile aroma compound synthesis, which impacts wine aroma profiles [50]. Cluster 10 includes grape varieties which are known for their distinctive aromatic characteristics. These grapes are traditionally cultivated in specific, often mountainous or hilly regions with unique terrain. The shared ASE signatures could reflect adaptation-linked gene expression controlling stress tolerance mechanisms, such as drought resistance or temperature resilience [51]. Many varieties in cluster 11 are known for producing wines with rich phenolic content, contributing to color, tannins, and antioxidant properties. Several cultivars in this group originate from Mediterranean or nearby regions, implying similar genetic responses to abiotic stresses [52]. The cultivars in cluster 12 are known for their strong aromatic profiles, especially muscat and floral notes. These varieties, often cultivated in regions with varying environmental pressures, might share allele-specific expression in stress-related genes [53]. Many of the grapes in cluster 13 are well known for their robust phenolic profiles that contribute to wine color, tannins, and aging potential. These varieties originate from various Mediterranean and Eastern European regions, suggesting shared genetic backgrounds influencing expression regulation [41]. Cluster 14 groups together grape varieties, which are known for their aromatic complexity and varying sugar accumulation during ripening [54]. Finally, the varieties in cluster 15 are typically known for their roles in producing light, aromatic white wines, often with delicate floral and fruity profiles [55].

### Comparison results

We then compared our result with those obtained (*i*) by using the *k*-means clustering algorithm [56] with squared Euclidean distance and considering each centroid as the mean of the points in a cluster, and (*ii*) by employing spectral clustering algorithm [13] where similarity graph has been obtained from the Euclidean distance. With the aim of assessing similarity among clusterings, we considered $k = 16$ for both algorithms and we employed Adjusted Rand Index (13). Table 2 shows that the results of the three algorithms are very different.

Since class labels are not available in our application scenarios, we resorted to *S-Dbw* index (9) as internal validation to assess the quality of the generated partitions. From last column of Table 2, it can be seen that, in our computational experiment, the minimum value was obtained by the proposed two-phase procedure. At this point, we would like to point out the effectiveness of splitting our procedure in two phases: the very high *S-Dbw* value of partition $\left\{ \mathcal {C}_{1}, \mathcal {C}_{2} \right\}$, obtained after the first phase, decreased significantly with the application of the second phase.

**Table 2:** Experimental results. Similarity among the results of the three clustering algorithms, computed by adjusted rand index, and goodness of partitions measured by means of *S-Dbw* index

	Two-phase clustering	*k*-means	Spectral clustering	*S-Dbw*
Two-phase clustering	1.000	− 0.012	− 0.023	0.415
*k*-means	− 0.012	1.000	0.062	2.581
Spectral clustering	− 0.023	0.062	1.000	3.377

For *k*-means and spectral clustering, the number of clusters obtained by applying our two-phase clustering has been used as input, namely, $k = 16$

After that, we compared the results of our algorithm with those obtained by employing *k*-means and spectral clustering, this time using CHI to determine the specific optimal number of partitions to both methods. According to it, the optimal number of clusters was 24. Even in this case, as reported in Table 3, we found that our approach outperforms the other ones.

**Table 3:** Experimental results. Similarity among the results of the three clustering algorithms, computed by adjusted rand index, and goodness of partitions measured by means of *S-Dbw* index

	Two-phase clustering	*k*-means	Spectral clustering	*S-Dbw*
Two-phase clustering	1.000	0.007	0.008	0.415
*k*-means	0.007	1.00	0.001	2.641
Spectral clustering	0.008	0.001	1	1.783

For *k*-means and spectral clustering, the optimal numbers of clusters have been computed by means of CHI

It is interesting to note that, when we moved from $k = 16$ to $k = 24$, the performance of *k*-means decreased. This could due to the *bias-variance trade-off*, where too many clusters can lead to overfitting the data, resulting in clusters that are too specific and do not generalize well to new data [57]. In addition, scalability should be considered as one of the major issues in clustering algorithms that can lead to drawbacks when faced with high-dimensional datasets [58]. In these cases, differences between data points can be indistinguishable, since many features become irrelevant. In other words, it can result that clusters are embedded in the subspaces of the entire feature space. Several clustering algorithms, including *k*-means, are not originally designed for high-dimensional data. Differently, our algorithm, as well as spectral clustering, make use of the spectrum (eigenvalues) of the similarity matrix of the data to perform dimensionality reduction before clustering in fewer dimensions, finding clusters within subspaces of the entire feature space. This is another point suggesting that our two-phase algorithm is more appropriate for clustering ASE dataset.

### Application to synthetic data

Simulation studies are a tool in statistics aiming to investigate the behaviour of a method when applied to synthetic data [59]. In our research line, these studies can be useful to compare different methods with the aim to determine which performs best in a given scenario, and to study the robustness of an algorithm if underlying assumptions are violated. For the problem of clustering a population of individuals starting from ASE analysis, benchmark dataset are not available. Therefore, to overcome this limit, we developed a simulator (paper in preparation) to generate synthetic expression matrix, as defined in Sect. 2, based on real data. The idea is to extend an expression matrix $\textbf{E} \in \mathbb {N}^{a \times m}$ with $m_{s}$ synthetic individuals that mimic the real data, obtaining a new expression matrix $\textbf{E}_{S} \in \mathbb {N}^{a \times (m+m_{s})}$.

Our generator is based on three main features: (*i*) the Hardy-Weinberg principle, which relates allele frequencies to genotype frequencies in a randomly mating population [60]; (*ii*) the effect of genetic operator in the evolution of the offspring [61]; (*iii*) a Markov Chain Monte Carlo method to extend the new population by generating individuals from a target distribution through an iterative update based on conditional probabilities.

**Fig. 2:**
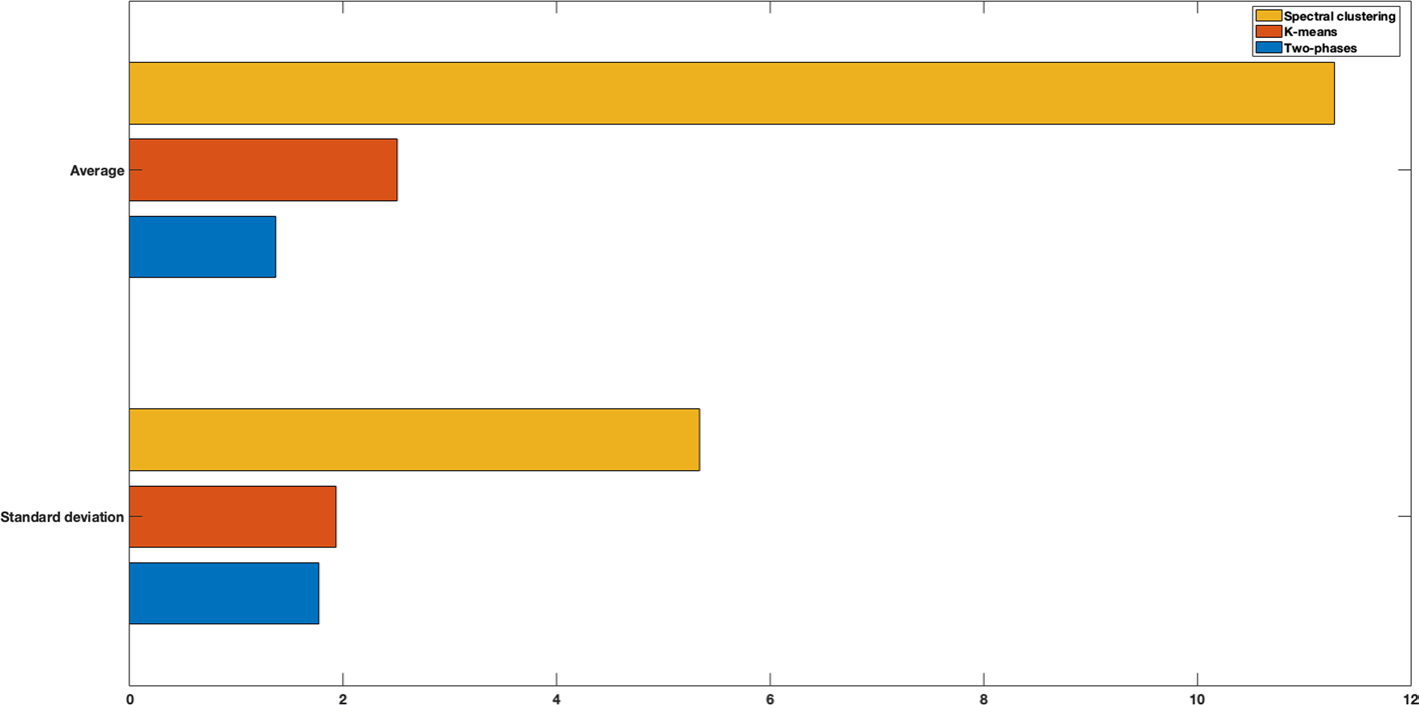
Clustering of synthetically generated populations. Goodness of partitions measured by means of *S-Dbw* index. Averages and standard deviations of *S-Dbw* index are represented for the two-phase clustering procedure, the *k*-means, and the spectral clustering algorithm

Starting from our expression matrix $\textbf{E} \in \mathbb {N}^{a \times m}$, where $m = 98$, we generated a new expression matrix $\textbf{E}_{S} \in \mathbb {N}^{a \times m^{'}}$, with $m^{'} = 10148$. We then created a set $\mathcal {P}_{S}$ of 50 synthetic populations by picking, in a random way, 100, 200, 300, 400, 500 columns of $\textbf{E}_{S}$. After that, we used our two-phase procedure, the *k*-means, and the spectral clustering to partition the populations belonging to $\mathcal {P}_{S}$. The results, by means of averages and standard deviations of *S-Dbw* index, are reported in Fig. 2. It is possible to see that our method outperforms the others.

### Evaluating the robustness of our algorithm

A robust clustering algorithm should be able to produce consistent and meaningful clusters regardless of the input data characteristics, such as the number, size, shape, and distribution of clusters, the presence of noise and outliers, and the choice of parameters and distance measures. Differently, a non-robust clustering algorithm may produce clusters that are sensitive to small changes in the data, that are arbitrary or meaningless, or that are influenced by irrelevant features.

We therefore evaluated the robustness of our algorithm by evaluating its ability to infer the partition $\mathcal {C}_{R}$ of real individuals even after extending the population with synthetic ones created by our generator.

First, we used as input of the two-phase algorithm the real expression matrix $\textbf{E} \in \mathbb {N}^{a \times m}$, where $m = 98$, to obtain the partition $\mathcal {C}_{R}$. Then the procedure has been applied 30 times to a new population obtained by expanding the real one with 100, 200, or 300 synthetic individuals, in order to obtain the partitions $\mathcal {C}_{S_{i}}$, $i=1,2,\ldots ,30$. Formally, we created a set $\mathcal {P}_{R \cup S}$ of 30 new populations by extending, in a random way, the real population with 100, 200, or 300 columns of $\textbf{E}_{S}$. After that, the Rand Index (RI) [62], limited to real individuals only, has been computed to evaluate the similarity between $\mathcal {C}_{R}$ and all the $\mathcal {C}_{S_{i}}$. We applied the same procedure also to *k*-means and spectral clustering, with the aim to compare their robustness to our algorithm’s. We applied, each time, *k*-means and the spectral clustering by employing CHI to determine the optimal number of partitions. To achieve a realistic simulation, we choose the optimal number of groups by optimizing the selected index within the interval [2, *m*/4] or $[2,m_{R \cup S}/4]$, where *m* is the number of individuals in the real population, while $m_{R \cup S}$ is the cardinality of $\mathcal {P}_{R \cup S}$.

Let us consider our population of *m* heterozygous individuals $P= \{p_1, \dots , p_m \}$. $\mathcal {C}_{R}$ represents the partition of *P* in *k* subsets. Let us suppose that $\mathcal {C}_{S_{i}}$ is a partition of a population of $\mathcal {P}_{R \cup S}$ into *s* clusters. Let us define: (*i*) *a* the number of pairs $(p_{i},p_{j}) \in P$ that are in the same cluster in both $\mathcal {C}_{R}$ and $\mathcal {C}_{S_{i}}$; (*ii*) *b* the number of pairs $(p_{i},p_{j}) \in P$ that are in different clusters in both $\mathcal {C}_{R}$ and $\mathcal {C}_{S_{i}}$; (*iii*) *c* the number of pairs $(p_{i},p_{j}) \in P$ that are in the same cluster in $\mathcal {C}_{R}$ and in different clusters in $\mathcal {C}_{S_{i}}$; (*iv*) *d* the number of pairs $(p_{i},p_{j}) \in P$ that are in different clusters in $\mathcal {C}_{R}$ and in the same cluster in $\mathcal {C}_{S_{i}}$. Then, RI is defined as:

14 $$\begin{aligned} RI = \frac{a+b}{a+b+c+d} = \frac{a+b}{m(m-1)/2}. \end{aligned}$$

**Fig. 3:**
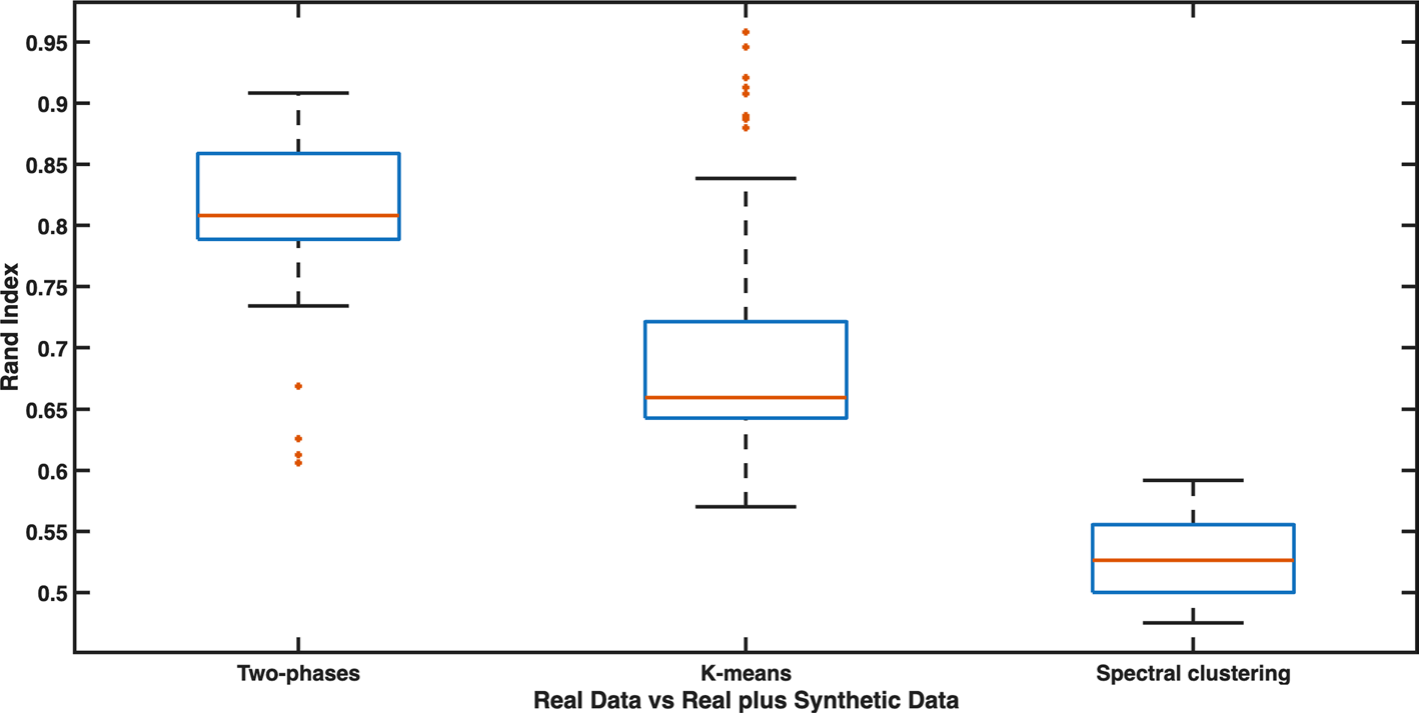
Evaluation of the robustness of our two-phase clustering algorithm. Boxplot showing the distribution of RI in our experiments. The central mark indicates the median, and the bottom and top edges of the box indicate the 25th and 75th percentiles, respectively. The whiskers extend to the most extreme data points not considered outliers, and the outliers are plotted individually using the * marker symbol. The figure also shows that the Two-phases procedure is more robust than *k*-means and spectral clustering

Since the numerator of (14) is the number of agreements between $\mathcal {C}_{R}$ and $\mathcal {C}_{S_{i}}$, while the denominator is the total number of pairs, RI represents the probability that $\mathcal {C}_{R}$ and $\mathcal {C}_{S_{i}}$ will agree on a randomly chosen pair: therefore, its value ranges between 0, indicating that the two data clusterings do not agree on any pair of points, and 1, indicating that the data clusterings are exactly the same. The boxplot in Fig. 3, displaying the distribution of RI in our experiments, reports a summary of five numbers, namely the minimum, first quartile, median, third quartile, and maximum, along with the outliers of the distribution. This shows that our clustering algorithm is able to re-produce the initial partitions in a consistent and meaningful way also when the population is extended with new synthetically generated individuals. Moreover, it is possible to observe that our method outperforms the *k*-means and the spectral clustering.

### CASTxMRL F1 hybrid mice dataset

To further validate our procedure, we considered the ASE dataset produced in [6]. In that work, three major cell populations (immune, fibroblast, and endothelial) are analyzed from hybrid crosses between two mouse strains, MRL (Murphy Roths Large) and CAST. While most mammalians heal injuries via the formation of fibrotic scar tissue, the MRL mouse can regenerate multiple tissue types without fibrosis. Due to this ability, MRL is dubbed a “super-healer", making it a valuable study subject. The authors collected samples from ear (which MRL can regenerate without fibrosis, unlike CAST) and dorsal (which both CAST and MRL heal by fibrosis) wounds of 3 F1 mice hybrids, on which bulk RNA-sequencing were performed and reads were mapped on F1 genome. Keeping CAST and MRL alleles separate, a total of 40 samples were obtained (16 immune, 12 endothelial and 6 fibroblast, each cell population being equally split by dorsal and ear tissue). Hierarchical clustering and principal component analysis (PCA) of ASE data, performed by the authors, grouped samples into distinct clusters by both wound site (ear-dorsal) and allele (MRL-CAST) for each cell population. This mice dataset thus appears to be a reasonable test set for our procedure, since its samples have a ground-truth labelling which could be determined by a clustering technique.

To evaluate whether our two-phase procedure can cluster samples by wound site and allele (4 ground-truth labels), we divided the 40 samples into 3 classes according to their cell population (16 immune, 12 endothelial and 12 fibroblast) and applied our method to one class at a time. In the following, we will refer to these classes as dataset1, dataset2, and dataset3, respectively. Next, we applied the procedure to all the 40 samples (dataset4), this time aiming to cluster them by wound site, allele, and cell population (12 ground-truth labels). Note that, in all these cases, we have only one allele per gene, although the actual expressed allele is different between the MRL and CAST samples.

As a second verification, we reworked the dataset by merging the MRL and CAST samples associated to the same cell population, wound tissue and individual (dataset5 in the following of the paper). Thus, each of the 20 new samples (8 immune, 6 endothelial and 6 fibroblast) has, for each gene, the details for both alleles. We applied the procedure to all the 20 new samples, this time aiming to cluster them by their wound site and cell population. (Results of the application of our two-phase clustering procedure to CASTxMRL F1 hybrid mice dataset can be found at the following URL: https://github.com/RobertoPagliarini/Two-phase-clustering-procedure/tree/main/CASTxMRL_dataset.)

**Fig. 4:**
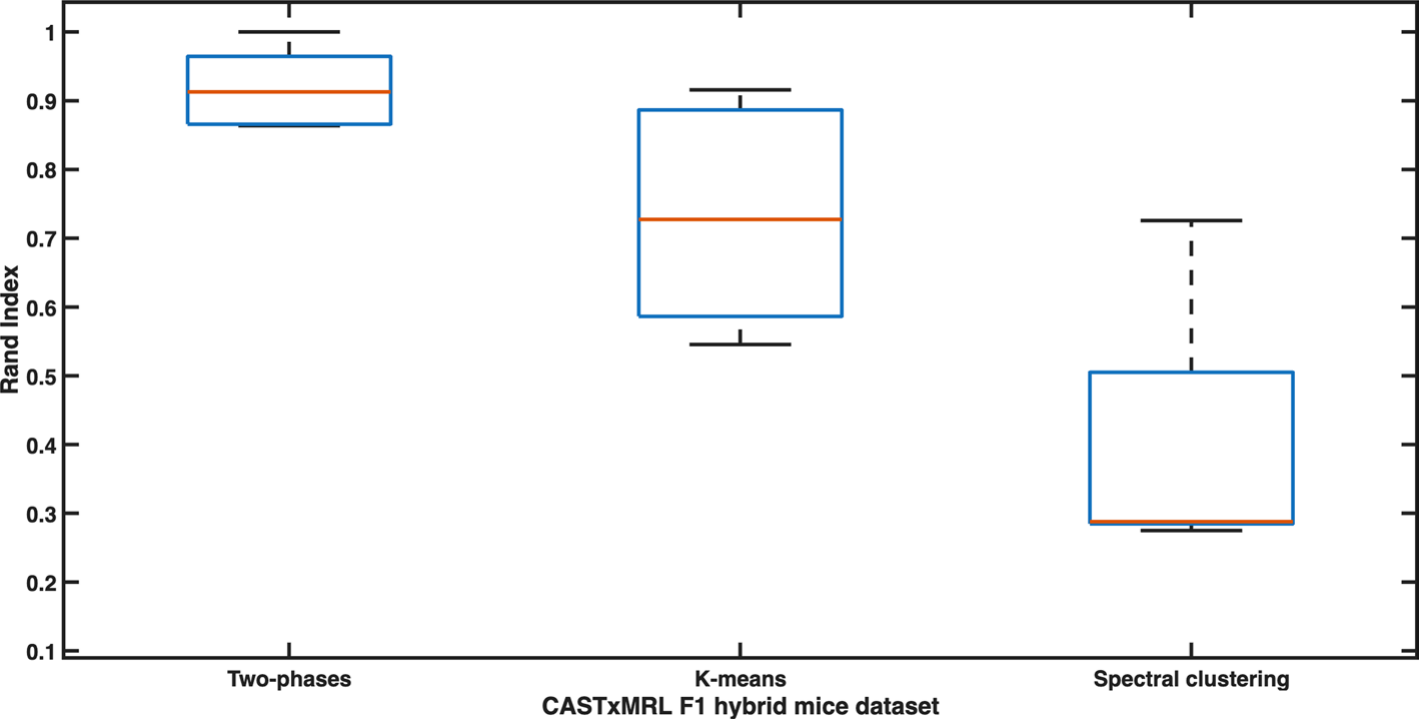
Boxplot showing the distribution of ARI in the experiments related to CASTxMRL F1 hybrid mice dataset. The central mark indicates the median, and the bottom and top edges of the box indicate the 25th and 75th percentiles, respectively. The whiskers extend to the most extreme data points not considered outliers, and the outliers are plotted individually using the * marker symbol

**Fig. 5:**
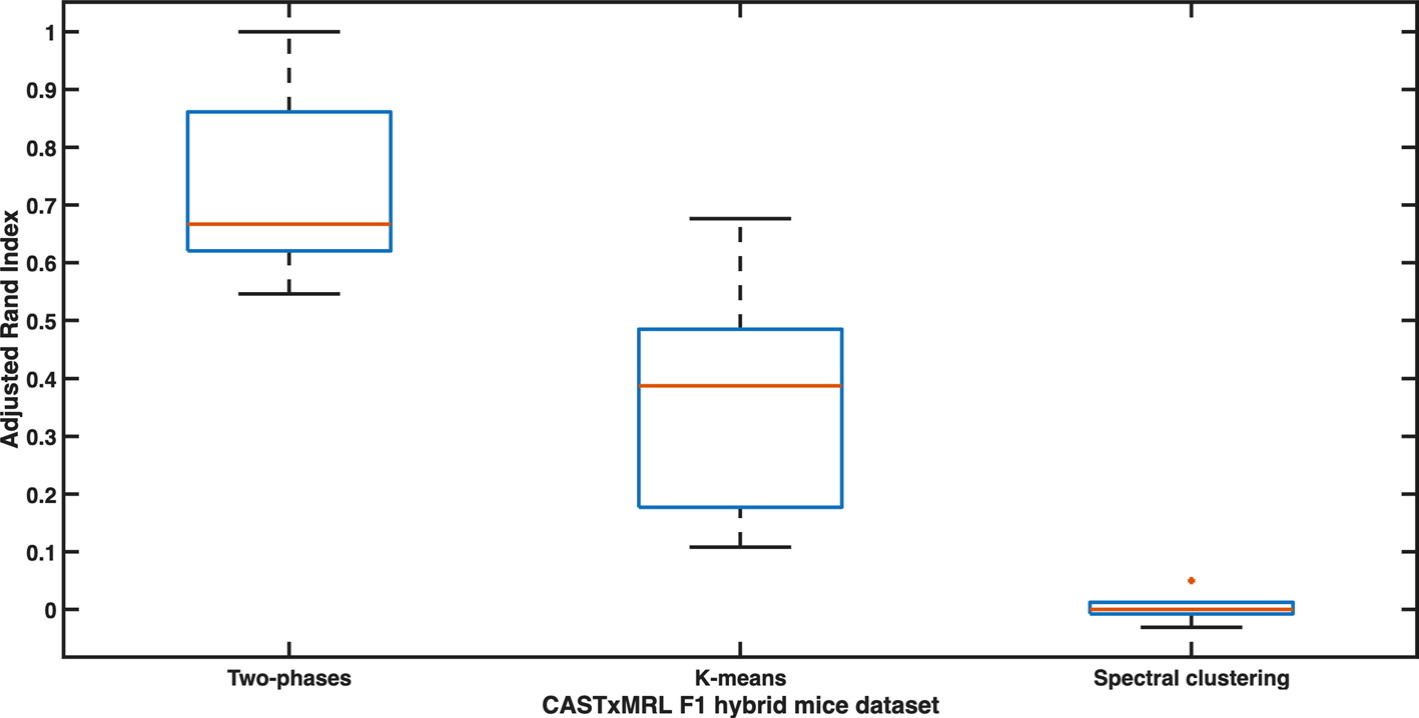
Boxplot showing the distribution of ARI in the experiments related to CASTxMRL F1 hybrid mice dataset. The central mark indicates the median, and the bottom and top edges of the box indicate the 25th and 75th percentiles, respectively. The whiskers extend to the most extreme data points not considered outliers, and the outliers are plotted individually using the * marker symbol

In order to compare our results, we partitioned the 5 datasets also by using *k*-means and spectral clustering. Also in this case, we employed CHI to determine the optimal number of partitions by following the steps used in previous sections. All the results, by means of RI and ARI, are reported in Table 4. It is possible to see how our algorithm outperforms others and reconstructs known clusters in a near-optimal way. Moreover, we can observe that the results are perfect for the dataset associated with the fibroblast population (dataset3), and quite optimal for the ones related to the whole 40-sample population (dataset4) and to the merged 20-sample population (dataset5). The distributions of RI and ARI for all the 5 dataset are graphically shown in Figs. 4 and 5, respectively.

**Table 4:** Clustering results on CASTxMRL F1 hybrid mice dataset. Goodness of inferred partitions, for two-phase clustering algorithm, *k*-means, and Spectral clustering, measured by means of RI and ARI

	Dataset1		Dataset2		Dataset3		Dataset4		Dataset5	
	RI	ARI	RI	ARI	RI	ARI	RI	ARI	RI	ARI
Two-phase clustering	0.867	0.668	0.864	0.645	1.000	1.000	0.913	0.546	0.953	0.815
*k*-means	0.600	0.200	0.546	0.108	0.727	0.421	0.877	0.388	0.916	0.676
Spectral clustering	0.2750	0	0.288	0	0.288	0	0.726	$-\,0.031$	0.431	0.050

Dataset1 = immune cells ASE dataset; Dataset2 = endothelial cells ASE dataset; Dataset3 = fibroblast cells ASE dataset; Dataset4 = full CASTxMRL F1 ASE dataset; Dataset5 = full merged CASTxMRL F1 ASE dataset

## Time complexity

Let us provide an insight on the time complexity of our method, that is a function of a dataset of *m* individuals, *a* considered alleles, and *k* clusters. Computing the similarity matrix, either in the first or second phase of the procedure, requires $O(am^{2})$ operations, as we need to compare each distinct pair.

With the aim to compute the smallest *k* eigenvalues and the corresponding eigenvectors, the *Lanczos algorithm* has been employed [19]. It requires $O(t) + O(t\textit{M}(m))$ time, where *t* is maximum number of matrix–vector computations required, while $\textit{M}(m)$ is the cost of a matrix–vector computation for solve the eigenvalue problem.

On the $m \times k$ matrix defined by the eigenvectors, the Lloyd’s algorithm takes time *O*(*ikam*), where *i* is the number of iterations until convergence [63]. With the *k*-means$++$ initialization, the algorithm is guaranteed to find a solution that is $O(\log k)$ competitive to the optimal *k*-means solution.

The complexity of *S-Dbw* index is based on those for the computation of two terms: the inter-cluster density that requires $O(amk^2)$ operations, and the intra-cluster variance which needs *O*(*amk*) operations. Usually $k \ll a$ and $k \ll m$, so the complexity of *S-Dbw* computation can be approximated as *O*(*amk*).

Finally, computation of the Calinski-Harabasz index requires time $O(m\log m)$. Differently, the time complexity of the Silhouette index computation is quadratic in the number of vectors involved in the clustering, whereas the time complexity of the Davies-Bouldin index computation is linear in the number of clustered vectors. We have also to consider that the computation of Dunn index for partitions of *m* individuals of *a* considered alleles has quadratic time complexity $O(am^2)$, so its computation is impractical for very large populations.

## Discussion

While many clustering tools have been proposed in the literature, there is no consensus on which methods are more suitable for real-world datasets. There is no such thing as a wrong-chosen algorithm: some is just more suitable for specific dataset structures, and some other for different ones. In the specific case of ASE analysis, data have very peculiar features and, in order to capture their underlying specificities, we introduced a matrix to store ASE starting from RNA-seq experiments and we proposed a novel two-phase clustering procedure to partition a population into groups of similar individuals. We illustrated the biological intuition behind it and we showed, by using both real case-studies and synthetic data, that our method outperforms other standard clustering algorithms, maintaining a high level of robustness.

In order to obtain better precision in our results, we are considering extending our algorithm with mechanisms to take into account allele frequency, i.e. the relative frequency of a variant of a gene at a particular locus in a population [64], and/or to integrate data on sequence distance between alleles. Clearly, the effectiveness of such extensions may depend on many parameters such as, for example, the size of the examined population.

Moreover, with the aim of validating our approach on a larger collection of different situations, we are developing a robust methodology to generate synthetic ASE datasets. Furthermore, we are planning to to carry out a detailed study in order to explore how different validation measures perform on our clustering results.

Finally, as a further experiment, in order to study *cis-regulatory* diversity at population level, we plan to apply the procedure to the ASE of the entire genome of all tissues present in grapevine variants dataset. The idea is to run the two-phase clustering procedure separately on all 19 chromosomes, yielding a new characterization of each individual, coding its cluster membership for each chromosome. Then, clustering could be exploited to identify homogeneous individual groups based on these vectors of features.

## Conclusions

In this work, we have defined an expression matrix capturing allele expressions from an RNA-sequencing experiment. On this matrix, we developed a novel two-phase unsupervised clustering procedure, built on top of a spectral clustering algorithm, whose aim is to partition the population into groups of similar individuals, according to their allelic expression. Using real case-studies as well as generated synthetic data, we proved that our algorithm shows significant robustness and outperforms other standard clustering techniques.

## Data Availability

Our software code is available at the following URL: https://github.com/RobertoPagliarini/Two-phase-clustering-procedure The results are available at the following URLs: https://github.com/RobertoPagliarini/Two-phase-clustering-procedure/tree/main/ResultsCIBB2024. https://github.com/RobertoPagliarini/Two-phase-clustering-procedure/tree/main/CASTxMRL_dataset The grapevine variants database can be found in [14]. The allele-specific count data (MRLxCAST) have been downloaded from the following repository: https://doi.org/10.6084/m9.figshare.c.6025157.

## Abbreviations

ARI: Adjusted rand index
ASE: Allele specific expression
CHI: Calinski–Harabasz index
DBI: Davies-Bouldin index
DUI: Dunn index
PCA: Principal component analysis
RNA-seq: RNA-sequencing
RI: Rand index
SHI: Silhouette index
